# Supplementary material for: Dynamic transcriptomic profiles of zebrafish gills in response to zinc supplementation
Source: BMC Genomics. 2010 Oct 11;11:553. doi: 10.1186/1471-2164-11-553 (PMC3091702; doi:10.1186/1471-2164-11-553)
Supplement: Additional file 2 — Interactive Direct Interaction Network representing the molecular interactions between zinc, copper, iron, calcium and proteins encoded by transcripts changed by zinc supplementation. Mini web-site containing index.html and hyperlinked pages in subdirectory describing a Direct Interaction Network automatically generated based on curated interactions contained within the proprietary PathwayArchitect database. Ovals represent proteins and the circles symbolize metal ions. Objects are coloured by their abundance in zebrafish at the time-point they were significantly different from the control is a scale from -4 fold (dark green) to +4 fold (dark red). Where significant differences were found at more than one time-point, the colour overlay shows expression at the first instance. Dark blue squares denote 'binding', and light blue squares 'expression'; green squares stand for 'regulation', green diamonds for 'metabolism', and green circles for 'promoter binding'. Arrow heads indicate directionality of the interaction where annotated. All nodes and edges can be further interrogated by selecting the relative area of the image. [file 1471-2164-11-553-S2.zip › PathwayArchitect Zn xs DIN/79300.html]

# PROTEIN: JUN

|  |  |
| --- | --- |
| Name | JUN |
| Type | PROTEIN |
| Description | v-jun sarcoma virus 17 oncogene homolog (avian) |
| Note | This gene is the putative transforming gene of avian sarcoma virus 17. It encodes a protein which is highly similar to the viral protein, and which interacts directly with specific target DNA sequences to regulate gene expression. This gene is intronless and is mapped to 1p32-p31, a chromosomal region involved in both translocations and deletions in human malignancies. |
| Alias | Jun activation domain binding protein |
|  | JUN |
|  | v-jun avian sarcoma virus 17 oncogene homolog |
|  | AP1 |
|  | Jun |
|  | p39 |
|  | c-jun |
|  | Avian sarcoma virus 17 (v-jun) oncogene homolog |
|  | Activator protein 1 |
|  | Proto-oncogene c-jun |
|  | immediate early gene |
|  | Junc |
|  | AH119 |
|  | enhancer-binding protein AP1 |
|  | AP-1 |
|  | Jun A |
|  | V-jun avian sarcoma virus 17 oncogene homolog |
|  | activator protein 1 |
|  | v-jun sarcoma virus 17 oncogene homolog |
|  | Rjg-9 |


---

|  |  |
| --- | --- |
| GO Component | nuclear chromosome |
|  | transcription factor complex |
|  | intracellular |
|  | nucleus |


---

|  |  |
| --- | --- |
| GO ID | GO:0005634 |
|  | GO:0003677 |
|  | GO:0000074 |
|  | GO:0006355 |
|  | GO:0050875 |
|  | GO:0005622 |
|  | GO:0035026 |
|  | GO:0003702 |
|  | GO:0000228 |
|  | GO:0005515 |
|  | GO:0045944 |
|  | GO:0005667 |
|  | GO:0006350 |
|  | GO:0003700 |
|  | GO:0008134 |


---

|  |  |
| --- | --- |
| MIM | MIM:165160 |


---

|  |  |
| --- | --- |
| Connectivity | 6528 |


---

|  |  |
| --- | --- |
| Entrez ID | 3725 |
|  | 24516 |
|  | 16476 |


---

|  |  |
| --- | --- |
| Agilent ID | A\_14\_P127903 |
|  | A\_23\_P201538 |
|  | A\_14\_P121894 |
|  | A\_14\_P109617 |
|  | A\_14\_P134030 |
|  | A\_51\_P325914 |
|  | A\_53\_P165892 |
|  | A\_42\_P741027 |
|  | A\_14\_P100102 |
|  | A\_53\_P143668 |


---

|  |  |
| --- | --- |
| Cellular Localization | Nucleus |
|  | Chromosome |
|  | Cell |
|  | Organelle |


---

|  |  |
| --- | --- |
| DbXref | KEGG pathway##04510##Focal adhesion##http://www.genome.jp/dbget-bin/show\_pathway?hsa04510+3725 |
|  | KEGG pathway##04620##Toll-like receptor signaling pathway##http://www.genome.jp/dbget-bin/show\_pathway?hsa04620+3725 |
|  | KEGG pathway##04310##Wnt signaling pathway##http://www.genome.jp/dbget-bin/show\_pathway?mmu04310+16476 |
|  | KEGG pathway##04310##Wnt signaling pathway##http://www.genome.jp/dbget-bin/show\_pathway?rno04310+24516 |
|  | KEGG pathway##04510##Focal adhesion##http://www.genome.jp/dbget-bin/show\_pathway?mmu04510+16476 |
|  | KEGG pathway##04660##T cell receptor signaling pathway##http://www.genome.jp/dbget-bin/show\_pathway?mmu04660+16476 |
|  | KEGG pathway##04010##MAPK signaling pathway##http://www.genome.jp/dbget-bin/show\_pathway?rno04010+24516 |
|  | KEGG pathway##04510##Focal adhesion##http://www.genome.jp/dbget-bin/show\_pathway?rno04510+24516 |
|  | KEGG pathway##04310##Wnt signaling pathway##http://www.genome.jp/dbget-bin/show\_pathway?hsa04310+3725 |
|  | KEGG pathway##04010##MAPK signaling pathway##http://www.genome.jp/dbget-bin/show\_pathway?mmu04010+16476 |
|  | KEGG pathway##04620##Toll-like receptor signaling pathway##http://www.genome.jp/dbget-bin/show\_pathway?rno04620+24516 |
|  | KEGG pathway##04662##B cell receptor signaling pathway##http://www.genome.jp/dbget-bin/show\_pathway?mmu04662+16476 |
|  | KEGG pathway##04010##MAPK signaling pathway##http://www.genome.jp/dbget-bin/show\_pathway?hsa04010+3725 |
|  | KEGG pathway##04620##Toll-like receptor signaling pathway##http://www.genome.jp/dbget-bin/show\_pathway?mmu04620+16476 |


---

|  |  |
| --- | --- |
| Pathway | IL-3 Signaling |
|  | PDGF Signaling |
|  | G-protein-MAPK Activation |
|  | Toll-like Receptor |
|  | SAPK-JNK Signaling |
|  | NGF Signaling |
|  | FAS Signaling |
|  | HIV |
|  | Erythropoietin Signaling |
|  | Insulin Signaling |
|  | IL-2 Signaling |
|  | Breast Cancer |
|  | Integrin Signaling |
|  | EGF Signaling |
|  | Zn xs inventory |
|  | Zn xs DIN |


---

|  |  |
| --- | --- |
| GO Process | leading edge cell differentiation |
|  | cellular physiological process |
|  | regulation of transcription, DNA-dependent |
|  | regulation of progression through cell cycle |
|  | transcription |
|  | positive regulation of transcription from RNA polymerase II promoter |


---

|  |  |
| --- | --- |
| UniGene | Mm.275071 |
|  | Hs.525704 |
|  | Rn.93714 |


---

|  |  |
| --- | --- |
| Affymetrix Probeset ID | 100130\_at |
|  | 1369788\_s\_at |
|  | 1374404\_at |
|  | 1389528\_s\_at |
|  | 1417409\_at |
|  | 1448694\_at |
|  | 171444\_r\_at |
|  | 1895\_at |
|  | 201464\_x\_at |
|  | 201465\_s\_at |
|  | 201466\_s\_at |
|  | 32583\_at |
|  | 89913\_s\_at |
|  | g12803622\_3p\_s\_at |
|  | g7710122\_3p\_a\_at |
|  | Hs.78465.0.S2\_3p\_a\_at |
|  | J04111\_at |
|  | Msa.4586.0\_s\_at |
|  | rc\_AA944014\_at |
|  | rc\_AA945867\_at |
|  | rc\_AI175959\_at |
|  | x12761\_s\_at |
|  | X17163cds\_s\_at |
|  | 213281\_at |
|  | AA393106\_at |
|  | RC\_D51060\_s\_at |
|  | TC19475\_at |
|  | TC36410\_at |


---

|  |  |
| --- | --- |
| GO Function | protein binding |
|  | RNA polymerase II transcription factor activity |
|  | transcription factor activity |
|  | DNA binding |
|  | transcription factor binding |


---

|  |  |
| --- | --- |
| Nucleotide | BC078738 |
|  | AK159220 |
|  | AK135023 |
|  | NM\_010591 |
|  | CR541752 |
|  | AK133722 |
|  | BU782554 |
|  | BC021888 |
|  | BC002646 |
|  | BC006175 |
|  | BC002081 |
|  | AK133867 |
|  | CD370800 |
|  | BC094032 |
|  | X12761 |
|  | BC009874 |
|  | X12740 |
|  | BC032086 |
|  | AY423853 |
|  | BT019759 |
|  | NM\_021835 |
|  | AK132019 |
|  | BI793062 |
|  | J04115 |
|  | AK159196 |
|  | NM\_002228 |
|  | J04111 |
|  | AL136985 |
|  | AY217548 |
|  | AK140911 |
|  | BG831943 |
|  | CR541724 |
|  | X17163 |
|  | X17215 |
|  | BC068522 |


---

|  |  |
| --- | --- |
| Protein | AAH78738 |
|  | AAH09874 |
|  | AAA37419 |
|  | BAE24518 |
|  | P05627 |
|  | P17325 |
|  | AAO22993 |
|  | CAA31252 |
|  | AAH02081 |
|  | AAH94032 |
|  | AAH21888 |
|  | AAH68522 |
|  | CAC10201 |
|  | NP\_068607 |
|  | AAA59197 |
|  | P05412 |
|  | AAR00344 |
|  | CAA35084 |
|  | BAE22389 |
|  | AAH06175 |
|  | CAG46552 |
|  | BAE34891 |
|  | NP\_002219 |
|  | BAE20944 |
|  | CAG46525 |
|  | CAA31236 |
|  | CAA35041 |
|  | NP\_034721 |
|  | AAV38564 |


---

|  |  |
| --- | --- |
| Organism | Mammal |


---

|  |  |
| --- | --- |
| Location | chromosome 5, 5q34.1 (Rattus norvegicus) |
|  | 4 44.6 cM (Mus musculus) |
|  | chromosome 4, 4 44.6 cM, 4 C5-C7 (Mus musculus) |
|  | chromosome 1, 1p32-p31 (Homo sapiens) |


---

|  |  |
| --- | --- |
